# Pathological and immunological analyses of *Thelohanellus kitauei* (Myxozoa:Myxosporea) infection in the scattered mirror carp, *Cyprinus carpio*

**DOI:** 10.1038/s41598-019-56752-w

**Published:** 2019-12-27

**Authors:** Tao Liu, Wen-Yan Wei, Kai-Yu Wang, Qian Yang, Er-Long Wang

**Affiliations:** 1 Department of Basic Veterinary, Veterinary Medicine College, Sichuan Agricultural University, Cheng’du, 611134 Sichuan P.R. China; 2 Key Laboratory of Animal Disease and Human Health of Sichuan Province, Sichuan Agricultural University, Cheng’du, 611134 Sichuan P.R. China; 3 China Institute of fisheries of chengdu agriculture and forestry academy, Cheng du, 610000 China

**Keywords:** Infectious-disease diagnostics, Infection

## Abstract

*Thelohanellus kitauei* is a spore-forming myxosporean parasite prevalent in scattered mirror carp (*Cyprinus carpio*) that generates numerous cysts in the intestine and causes mass mortality in fish. To investigate the infection and mortality induced by *T. kitauei in* pond-reared farms in Luo-Jiang (104°51’N, 31°31’E), southwest China, morphological and molecular analyses of infected fish were conducted. Natural and specific immune indicators were further evaluated to determine the immunological effects of response to parasitic infection. The infectious parasite was identified as *Thelohanellus kitauei* based on morphological, 18S rDNA and infectious characteristics. Scattered mirror carp was determined as the specific intermediate host of the parasite. However, *T. kitauei* still caused considerable damage to the fish, in particular, injury and blockage of the intestines, resulting in malnutrition and even death. The mature spores of *T. kitauei* colonize the intestinal submucosa of carp and form cysts of various sizes that block the intestinal tract and release spores into the enteric cavity upon rupture, leading to the next phase of *T. kitauei* growth. Moreover, *T. kitauei*-infected carp showed weaker innate immunity. IgM is involved in the fight against parasitic infection while cytokines, such as IL-6, IL-1β and TNF-α, had an impact on infection processes. To our knowledge, this is the first report to show that *T. kitauei* infects and causes death in scattered mirror carp. Our collective findings from systematic pathology, morphology and immunology experiments provide a foundation for further research on infections by this type of parasite and development of effective treatment strategies.

## Introduction

Microscopic Myxosporea (clade Myxozoa) belonging to the phylum Cnidaria is a widespread subclass (2,200 species) in the marine and freshwater environments^1^. While Cnidarians are generally regarded as free-living animals^2^, it is now apparent that some species of Myxozoa are important endoparasites of fish^1^. Emerging diseases caused by certain myxosporea are usually associated with environmental changes, such as the impact of intensive culture of fishery industries worldwide^3–7^. Myxozoan infections have become more frequent, resulting in mass mortality in fish farms and severe economic losses to aquaculture^7–11^.

Considerable literature on myxosporea has been published to date. In general, marine myxosporeans have a wide host range. For instance, *Kudoa yasunagai*^12,13^ can infect *Lateolabrax japonicus*, *Oplegnathus fasciatus*, *Seriola quinqueradiata*, *Takifugu rubripes*, *Thunnus orientalis* and *Plotosus lineatus*, forming numerous cysts in the brain and causing disorders in swimming performance. Recent studies have shown that myxosporeans of fish are mostly host-specific parasites, typically infecting only one host species (oioxenic) or a limited number of closely related species (stenoxenic)^1,4,9,10^. In fact, the majority of freshwater myxosporeans are oioxenic or stenoxenic species. *Myxobolus murakamii*^14^, the agent of myxosporean sleeping disease in salmonid fish, infects nerve tissues. *Myxobolus artus*^15^, the causative agent of muscular myxobolosis, has a fatal effect on farmed common carp *Cyprinus carpio*, along with *Thelohanellus hovorkai*^16,17^, the causative agent of hemorrhagic thelohanellosis.

In China, over 600 species of myxozoan parasites infecting various cultured fish have been identified, the majority causing significant economic losses in aquaculture^18^. In particular, intestinal giant cystic disease caused by the myxozoan species *Thelohanellus kitauei*^18^ is the most detrimental disease of carp, the most commonly farmed fish species in China. Around 20% farmed carp die annually due to this disease, leading to an economic loss of ~50 million dollars every year^18,19^. Historically, *T. kitauei* infection of common carp has been reported in a number of East Asian countries, mainly Japan, Korea, and China^19^, but not the Americas, although the common carp is globally distributed^19,20^. In addition, The developing stage of *T. kitauei* has been found in the *Branchiura sowerbyi* in Hungary^21^, indicating that the myxospores of *T. kitauei* existed in Hungary (or European) ecosystem, but the stages in the fish-intermediate host hasn’t been found as yet.

In July 2018, mass mortality of pond-reared scattered mirror carp was reported from fish farms in southwest China. Examination of diseased fish revealed accumulation of giant cysts in the gut. In the present study, a *Thelohanellus* species with similar spore morphology to *Thelohanellus kitauei* was isolated from intestinal cysts of scattered mirror carp (*C. carpio*) from carp farms. The two primary aims of this investigation were to: 1. identify the species of infecting parasite and 2. establish immunity to the parasitic infection and pathological effects on the host. We performed morphological, molecular, innate and adaptive immunity, and histological evaluation of infected tissues, with a view to clarifying the infectious cycle of the pathogen and developing effective control measures to manage or prevent future disease outbreaks.

## Materials and Methods

### Ethics statement

All animal experiments were approved by the Committee of Ethics on Animal Care and Use of Sichuan Agricultural University (No. XF201418). Experimental procedures were performed in accordance with the guidelines for care and use of experimental animals of the Chinese Ministry of Science and Technology.

### Sampling

In total, 60 fish were collected from a pond in Luo-Jiang Town (104°51’N, 31°31’E), De-Yang City, Sichuan Province, southwest China, during September 2018. The 20 000 m^2^ pond was stocked with 80 000 scattered mirror carp (*Cyprinus carpio*). After disease outbreak, the mortality rate was as high as 30%, with no sign of alleviation. Dissection of diseased fish revealed the presence of a large number of cyst-like hyperplasia in the intestinal tract composed of numerous spores, which led to death in fish. A total of 60 infected and 60 uninfected fish in the same pool were used for study. Six infected and six uninfected fish were analyzed for blood count and the remaining fish from both groups tested for serum immunity. Tests for each serum immune indicator required six fish from both infected and uninfected groups. A total of nine serum immune indicators were evaluated in 54 infected and 54 uninfected fish. Analysis of Serum immune indicators requires a large number of host fish owing to the difficulty of serum sampling in fish. Histological and parasitic samples were obtained from six random fish of the infected group and sampling in this area did not interfere with sampling of the above indicators.

### Morphological examination

Morphological and histological examinations were based on the method of Ye *et al*.^19^, with the following slight modifications: intestinal tracts were isolated from the internal organs of diseased fish through careful dissection. Fresh spores were removed from intestines of diseased fish and placed on a glass slide, compressed and treated with 10% NaOH solution for 1 min. Images were obtained under an optical microscope (olympus, Japan). Spores were further stained with crystal violet for 30 s and photographed under a light microscope.

### Histological examination

The diseased tissue was fixed in 10% neutral formaldehyde for histological observation. Further, the tissue was embedded in paraffin, cut into 3 μm sections, and stained with hematoxylin and eosin and observed under a light microscope (olympus, Japan). Another part of the diseased tissue needs to be cut into small pieces of 1 cm^3^ and fixed with 2.5% glutaraldehyde solution. Then, it is washed 3 times with 0.65% physiological saline, fixed with 1% OSO4 for 1 h, continuously dehydrated with acetone and dried. Finally, it was observed under a scanning electron microscopy (SEM). The tissue infected with Myxospores was directly observed under a light microscope (olympus, Japan) after being compressed.

### Molecular analysis

We employed the research methods of Tong *et al*.^19^ with minor modifications, as follows: cysts were isolated from infected intestinal tracts of *C*. *carpio* and preserved in 95% ethanol for molecular analyses. Several fragments of individual cysts were obtained and placed in 1.5 mL Eppendorf tubes. After ethanol evaporation, cyst fragments were ground in tubes with glass pestles. Total DNA was extracted using a Tissue/Cell Genomic DNA Extraction Kit (TIANGEN, China) according to the manufacturer’s protocol. The 18S rDNA gene was amplified with the primer pairs F1: 5′-CTGCGGACGGCTCAGTAAATCAGT-3′ and F2: 5′-CCAGGACATCTTAGGGCATCACAGA-3′. Polymerase chain reaction (PCR) was performed in a 25 μL reaction consisting of 12.5 μL 2 × PCR Mixture (Takara, China), 8.5 μL water, 2 ng each primer, and 15 ng isolated DNA. Next, DNA was denatured at 98 °C for 3 min, followed by 32 cycles at 95 °C for 45 s, 56 °C for 60 s, and 72 °C for 90 s, with final extension at 72 °C for 12 min. Amplified products were purified using a PCR Purification Kit (TIANGEN, China) according to the manufacturer’s protocol. A Basic Local Alignment Search Tool (BLASTn) search was performed to compare newly obtained sequences with highly similar sequences in the NCBI database. Consequently, sequences of a total of 47 taxa related to *Thelohanellus kitauei* were selected for alignment. Evolutionary history was inferred using the Maximum Likelihood method based on the Tamura-Nei model^22^. The tree with the highest log likelihood (-9951.87) is depicted. The percentage of trees in which associated taxa clustered together is shown next to the branches. Initial tree(s) for the heuristic search were obtained automatically by applying Neighbor-Joining and BioNJ algorithms to a matrix of pairwise distances estimated using the Maximum Composite Likelihood (MCL) approach and selecting topology with a superior log likelihood value. The tree is drawn to scale, with branch lengths measured in the number of substitutions per site. The analysis involved 47 nucleotide sequences. All positions containing gaps and missing data were eliminated. A total of 1113 positions were included in the final dataset. Evolutionary analyses were conducted in MEGA7^23^.

### Automatic whole blood count

Six individuals (weighing about 500 g) from both infected and non-infected groups were anesthetized with MS-222 and tail vein blood collection performed using a disposable medical syringe. About 150 μL blood per fish was used for blood cell sorting and the remaining sample for preparing serum. Blood samples were primarily used to detect white blood cell (including total white blood cells, lymphocytes and eosinophils) and red blood cell counts. Blood cell assays were performed on the XFA6100 automatic hematology analyzer (PuLang, Beijing).

### Measurement of innate immune parameters

Complement C3, complement C4, alkaline phosphatase, immunoglobulin IgM, immunoglobulin IgG, and superoxide dismutase (SOD) activity were measured to evaluate innate immune and specific immune responses using commercial kits according to the manufacturer’s instructions (Nanjing Jiancheng Bioengineering Institute, Nanjing, China). For quantification of IL-6, IL-1β and TNF-α levels in culture supernatant fractions, ELISA (Jiangsu Meimian industrial Co., Ltd) was performed.

### Statistical analysis

Statistical analysis was performed using GraphPad Prism 8^24,25^ software and differences among groups detected with one-way analysis of variance (ANOVA). In all cases, the significance level was defined as *P* < 0.01, and results presented as means ± SE (standard error).

## Results

### Host symptoms and characterization of cysts

**Figure 1:**
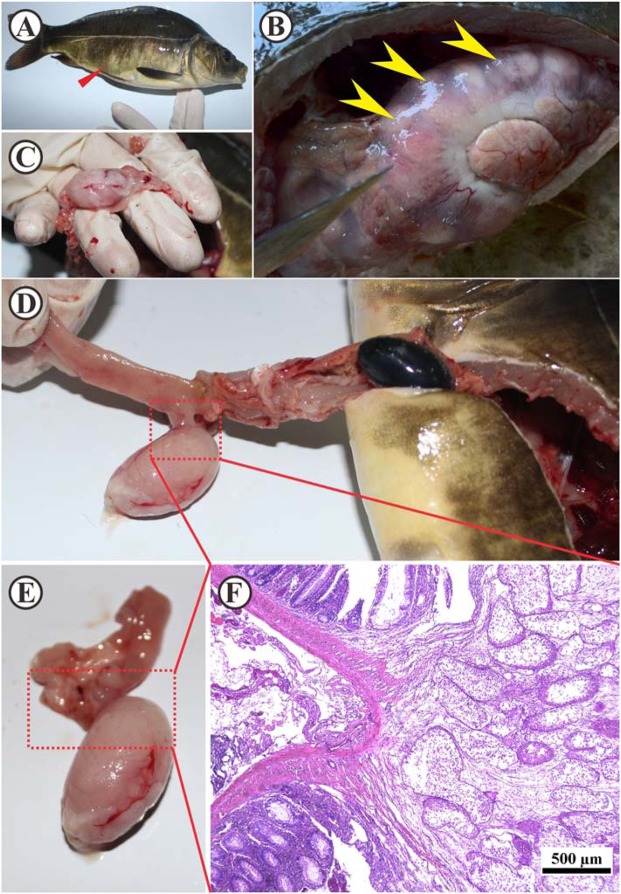
Gross presentation of infected *Cyprinus carpio* with macroscopic cysts in the intestine. (**A**) Infected scattered mirror carp (*Cyprinus carpio*) with swollen abdomen (red arrow). **(B)** Abdominal anatomy of infected carp: numerous cysts (yellow arrows) caused swellings that blocked the intestinal lumen in carp; **(C**–**E)**. The cyst was in direct contact with intestinal mucosa. **(F)** Cross-section of cyst within the dotted box in D and E (H&E stain, bar = 500 µm).

Diseased scattered mirror *C*. *carpio* ranging from 500 to 1000 g in weight were collected from a fish pond in Sichuan Province, China, in April 2018. Infected fish showed ataxia, unresponsiveness, swimming weakness, and even dysfunction such as lethargy and anorexia. In addition, the diseased fish showed a characteristic of abdominal enlargement (Fig. 1A).

Anatomic observation of the diseased fish revealed that the intestinal wall was thinned due to the cyst formed by the myxospores, and ascites appeared in the abdominal cavity (Fig. 1B,C). No food was observed in the intestines. From the intestines infected with fish, many cysts of different sizes (about 15–50 mm in size) can be obtained (Fig. 1D–F).

### Light microscopy

**Figure 2:**
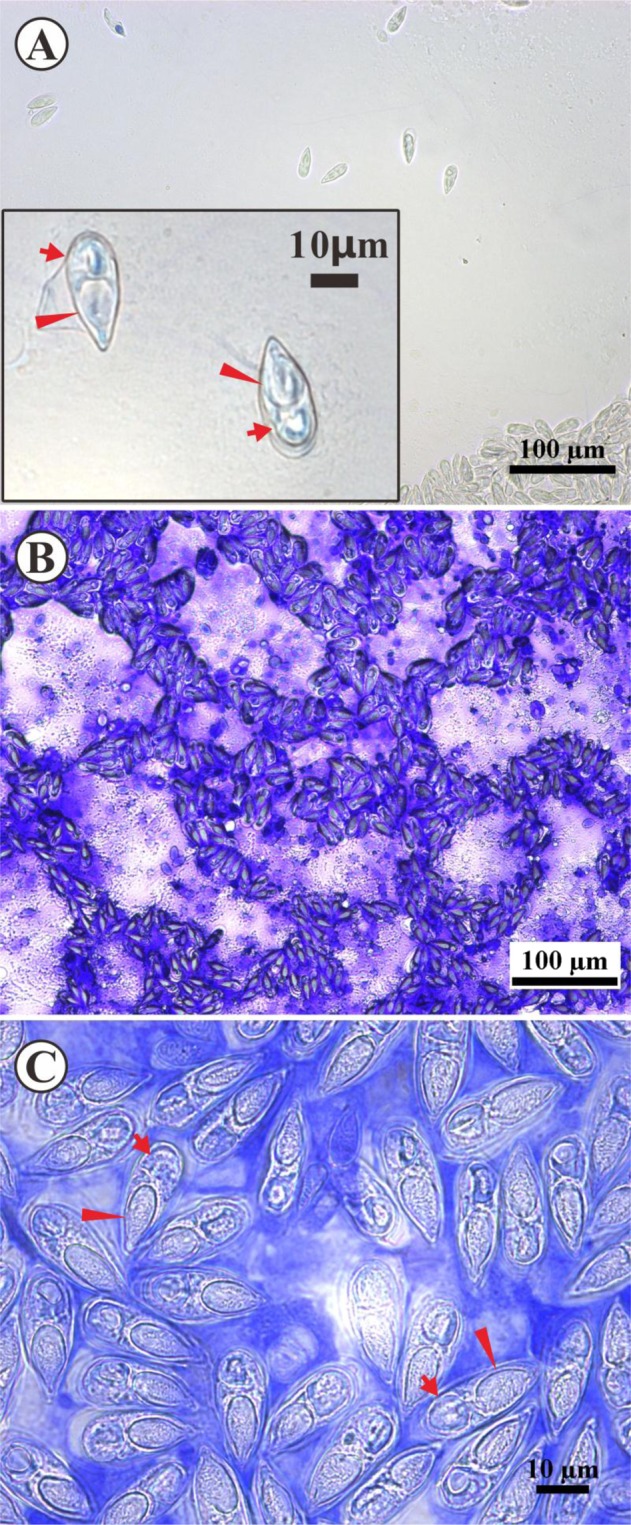
Spore morphology of *Thelohanellus kitauei* from cysts of *Cyprinus carpio*. (**A**) Light micrograph of a wet-mount section of excised cyst from the intestine. *Thelohanellus kitauei* spore containing a polar capsule (triangle) and sporoplasm (arrow). Scale bar = 10 µm/100 µm. (**B**) Fresh spores stained with 1% crystal violet solution. Scale bar io100 μm. (**C**) Fresh spores stained with 1% crystal violet solution, polar capsule (triangle) and sporoplasm (arrow). Scale bar = 10 µm.

The hyperplasia tissue in the intestinal tract of diseased fish was tableted on a glass slide, and after being transparent by sodium hydroxide, numerous spores were observed with a length of ~30 μm and width of ~10 μm (Fig. 2A). To further establish the morphological structure of spores, we conducted crystal violet staining on the pressed tablets. After staining, spores were dark blue in color (Fig. 2B). Following a short period of staining, internal structures of the spores were difficult to observe under the light microscope due to absorption of some of the pigments (Fig. 2B). Consequently, we re-treated the stained slides with sodium hydroxide, which allowed clearer observation of the partial spore structure under the light microscope. The mature spore shell was melon-like with a slightly pointed front end and rounded back (Fig. 2C). Two capsules (polar pockets) were clearly detected within the spores (Fig. 2C). Single polar capsules were pyriform and situated at the anterior extremity of the spore, occupying over half the length of the spore body (Fig. 2C). A filamentous hollow tubular structure was observed in the upper capsule (Fig. 2C). The majority of spores were surrounded by a membranous sheath (Fig. 2C).

### Histopathology

**Figure 3:**
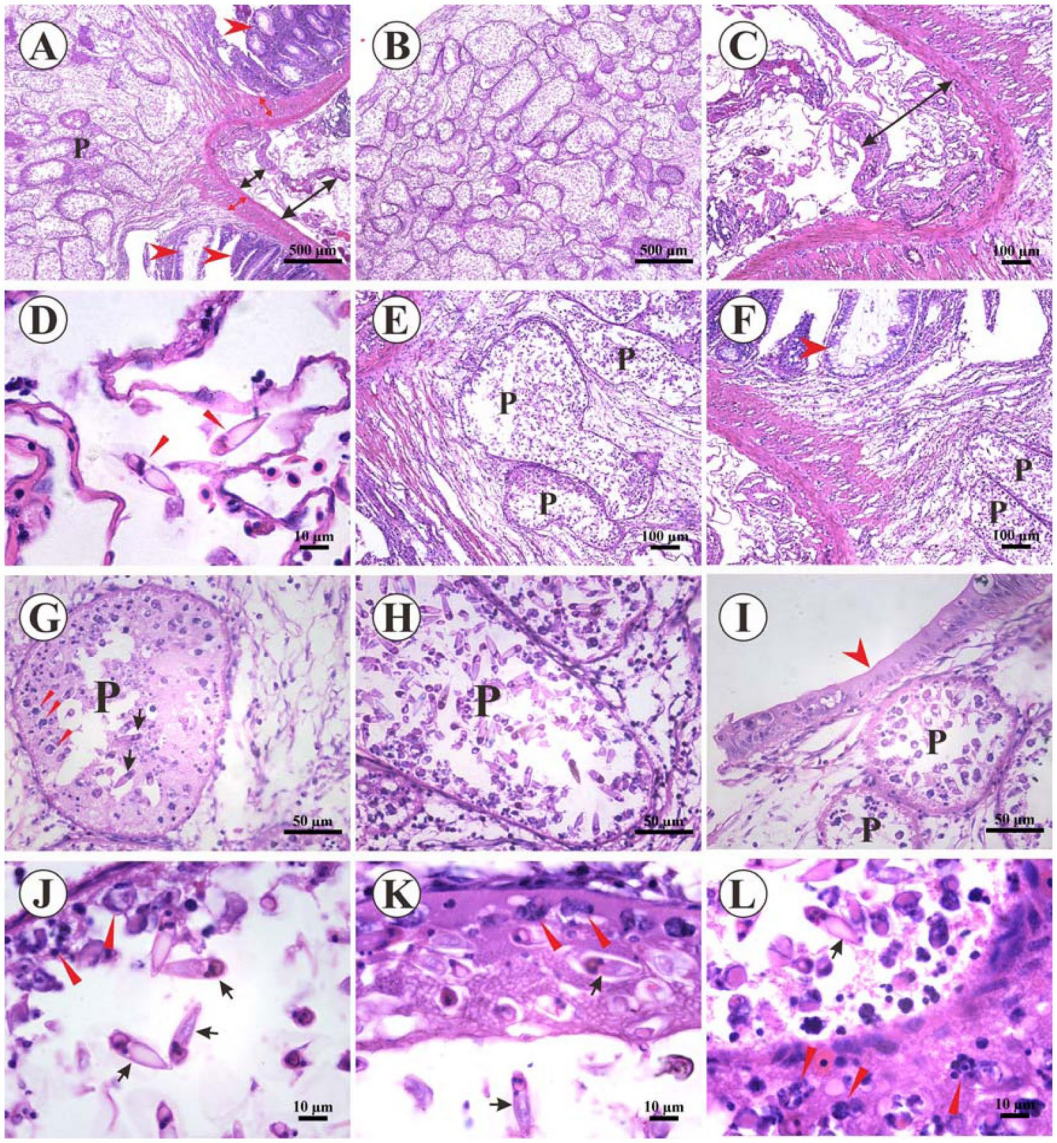
Histological lesions in *Cyprinus carpio* infected with *Thelohanellus kitauei* (*H&E staining*). (**A**) *Cross-section of the cyst in the dotted box from* Fig. 1D,E. Numerous *Thelohanellus kitauei* plasmodia (P) in intestine of *Cyprinus carpio*, submucosa of the intestine (red two-way arrow), serosa of the intestine (black two-way arrow), intestinal villus (red arrow), bar = 500 µm. (**B**) Numerous plasmodia *in T. kitauei* cysts. Scale bar = 500 μm. **(C)** Edema in intestinal serosa (black two-way arrow). Scale bar = 100 μm. **(D)** Spores in intestinal serosa (red arrowhead). Scale bar = 10 μm. **(E**) Intestinal mucosa replaced by *T. kitauei* plasmodia (P). Scale bar = 100 µm. **(F**) Fibrous hyperplasia around the *T. kitauei* cyst, *T. kitauei* plasmodia (P). Scale bar = 100 µm. Epithelial layer (red arrowhead). **(G**) Developing trophozoites (red arrowhead) of *T. kitauei* and spores within plasmodia of the cyst. Scale bar = 50 µm. **(H**) Several *T. kitauei* spores in plasmodia (P). Scale bar = 50 µm. **(I)** Developing plasmodia (P) under intestinal epithelial cells (red arrow). Scale bar = 50 µm. **(J/K/L)**. Developing trophozoites (red arrowhead) at the edge of plasmodia and mature spores in the central region of plasmodia (black arrow). Scale bar = 10 μm.

To elucidate the mechanism by which T. kitauei infects fish, intestinal histopathological analyses were performed. We observed attachment of the entire parasite cyst to the intestinal wall. Specifically, the capsule expanded from the basal layer into the intestine (Fig. 3A) and intestinal villi were “squeezed” to both sides (red arrow) (Fig. 3A). Significant edema of the serosal layer of the intestine was evident (Fig. 3A,C). A small number of T. kitauei were scattered in edema of the serosa (Fig. 3D). Numerous small capsules (P) of different sizes were present within the entire large capsule in the intestine (Fig. 3B,E). The intestinal mucosa at the site of infection was almost completely replaced by cysts while the uninfected part of intestinal villi remained almost intact (Fig. 3F). We detected a large number of trophozoites and parasites in the small capsule (Fig. 3G,H). Further extensive examination revealed that the entire large cyst was located within intestinal villi and had a tendency to break through villi, leading to rupture of a marginal part of the intestine (Fig. 3I). Therefore, it is speculated that during the initial period, the cyst gradually proliferates into the intestinal tract in the basal layer of the intestine. Upon proliferation to a certain size, intestinal villi form a separate capsule that completely falls into the intestinal lumen. A separate capsule may be further excreted with feces in the subsequent life cycle. This finding is in line with our anatomical observations. Additionally, in the small capsule, larvae of these monopoles appeared to develop from the edge of the small cyst, and increasing maturity of the pathogen was associated with closer proximity to the central part of the small capsule (Fig. 3J–L).

### Scanning electron microscopy

**Figure 4:**
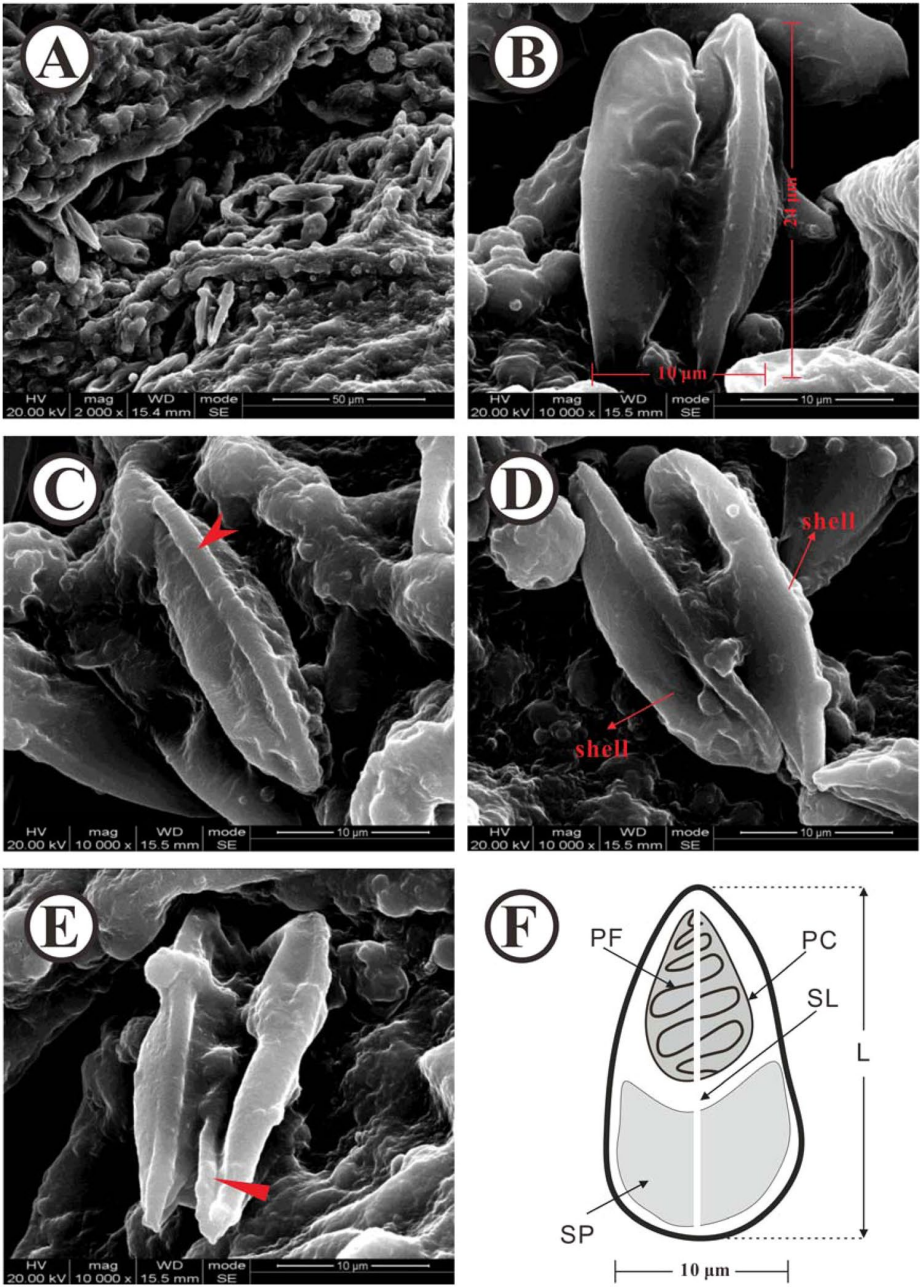
Scanning electron micrographs of *Thelohanellus kitauei* from intestines of scattered mirror carp *Cyprinus carpio*. (**A**) Numerous *T. kitauei* of the intestine, bar = 50 µm. (**B**) The morphology of the *T. kitauei*. Scale bar = 10 μm. (**C**) The prominent ridges of the *T. kitauei (*red arrowhead*)*. Scale bar = 10 μm. **(D)** Two shells of the *T. kitauei* (red long arrowhead). Scale bar = 10 μm. **(E)** The filament of the *T. kitauei (*red arrowhead*)*. Scale bar = 10 µm. **(F)** Structural patterns of the *T. kitauei*. Scale bar = 10 µm.

To further clarify parasite structure, infected intestine was examined via scanning electron microscopy. Microscopic observations revealed a large number of spores attached to submucosa of the intestine (Fig. 4A), consistent with histological observations. Spore surface was smooth, with no patterns and less wrinkles (Fig. 4B). Spores were about 24 μm long and 10 μm wide (Fig. 4B) with prominent ridges and a thin, straight suture on the ridge (Fig. 4C). The front ends of the two spores were asymmetrical in shape, with one end displaying a smooth front and no protrusion and the other end with a protruding structure (Fig. 4D). The filament surface was smooth and protruded from the front end of the structure inside the pathogen body (Fig. 4E). Based on collective histological and scanning electron microscopy findings, we plotted structural patterns of the parasite (Fig. 4F).

### Molecular characteristics and phylogenetic analysis

**Figure 5:**
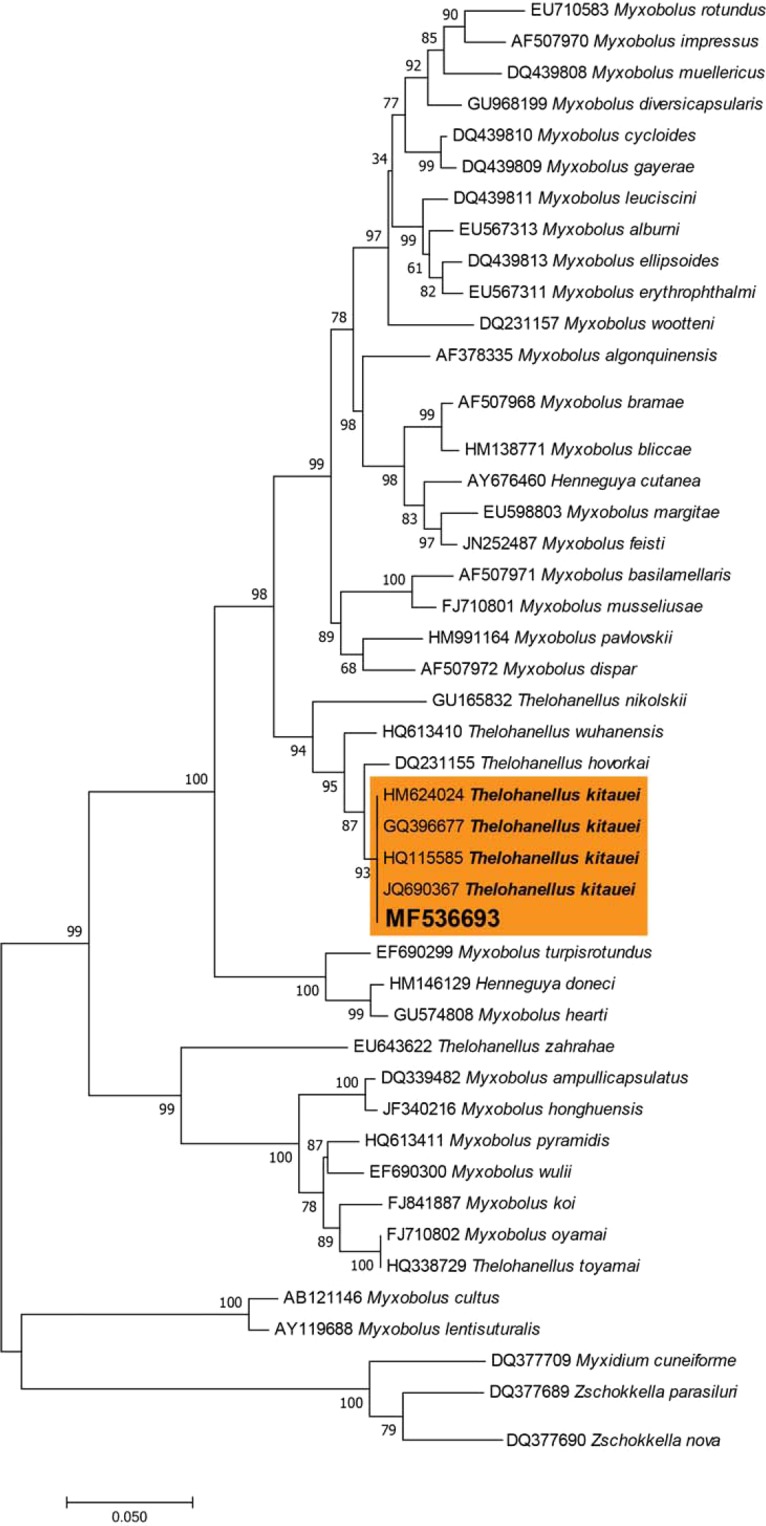
Molecular phylogeny analysis (Maximum Likelihood method) based on 18 rDNA sequences of myxosporeans. Numbers at nodes indicate bootstrap confidence values (1,000 replicates).

Morphological characteristics and infection specificity did not provide sufficiently comprehensive data to facilitate identification of the parasite isolated from carp and further analysis at the molecular biology level was required. Accordingly, we amplified the 18 S rDNA sequence with a length of 1547 bp., which was subsequently uploaded to NCBI (GenBank accession number, MF536693). BLAST results showed that the isolated parasite showed the highest sequence similarity with *Thelohanellus kitauei* [KU664644] and *Thelohanellus kitauei* [JQ690367] in GenBank (99.81% identity). In phylogenetic analysis, *Myxobolus* was divided into two clusters by monopoles and did not form completely independent branches (Fig. 5). The species represented by the MF536693 sequence clustered with *Thelohanellus kitauei* (Fig. 5). Morphological comparisons and molecular biology analyses led to the identification of the isolated parasite as *Thelohanellus kitauei*.

### Effects of *Thelohanellus kitauei* on immune cells of infected fish

**Figure 6:**
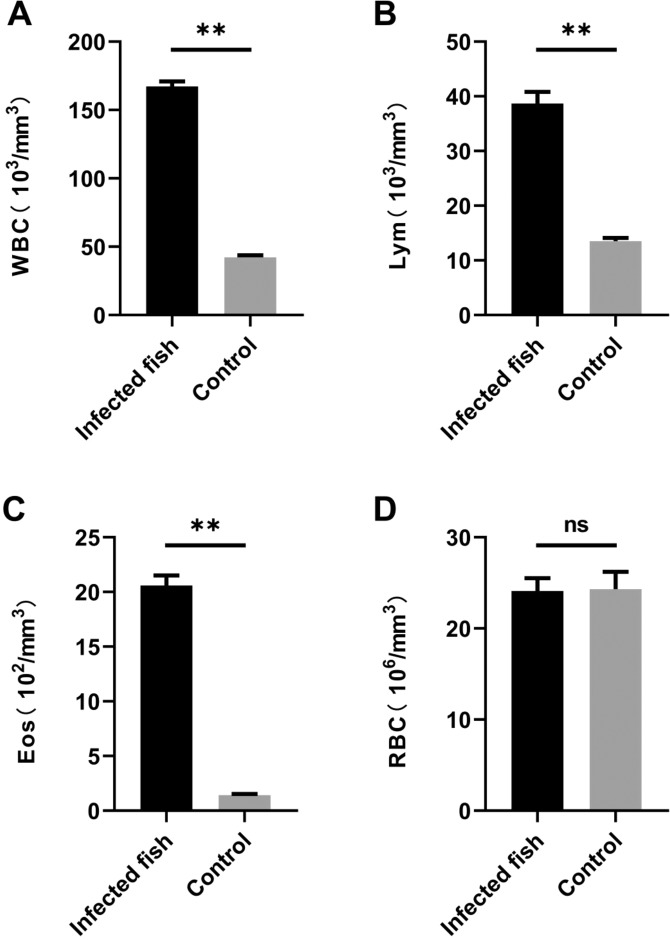
Routine blood examination of scattered mirror carp. Non-infected scattered mirror carp from the same pond were used as a control group. WBC, white blood cell count; Lym, lymphocyte count; Eos, eosinophil count; RBC, red blood cell count.

Immunological analyses are critical for understanding the characteristics of parasitic infections. Since *T. kitauei* did not parasitize parenchymal organs (such as liver, spleen and kidneys), but infected intestines of fish, we deduced that changes in peripheral immune cells could be detected in blood samples. Our findings suggest that *T. kitauei* infection triggers activation of cellular immunity in fish. The total counts of white blood cells (WBC) (P < 0.01) (Fig. 6A), lymphocytes (Lym) (P < 0.01) (Fig. 6B) and eosinophils (Eos) (P < 0.01) (Fig. 6C) of infected fish were markedly increased, compared to those of uninfected carp from the same pond. However, we observed no significant differences in red blood cell counts between the infected and uninfected groups of fish (Fig. 6D).

### Effects of *Thelohanellus kitauei* on the serum immune index of infected fish

**Figure 7:**
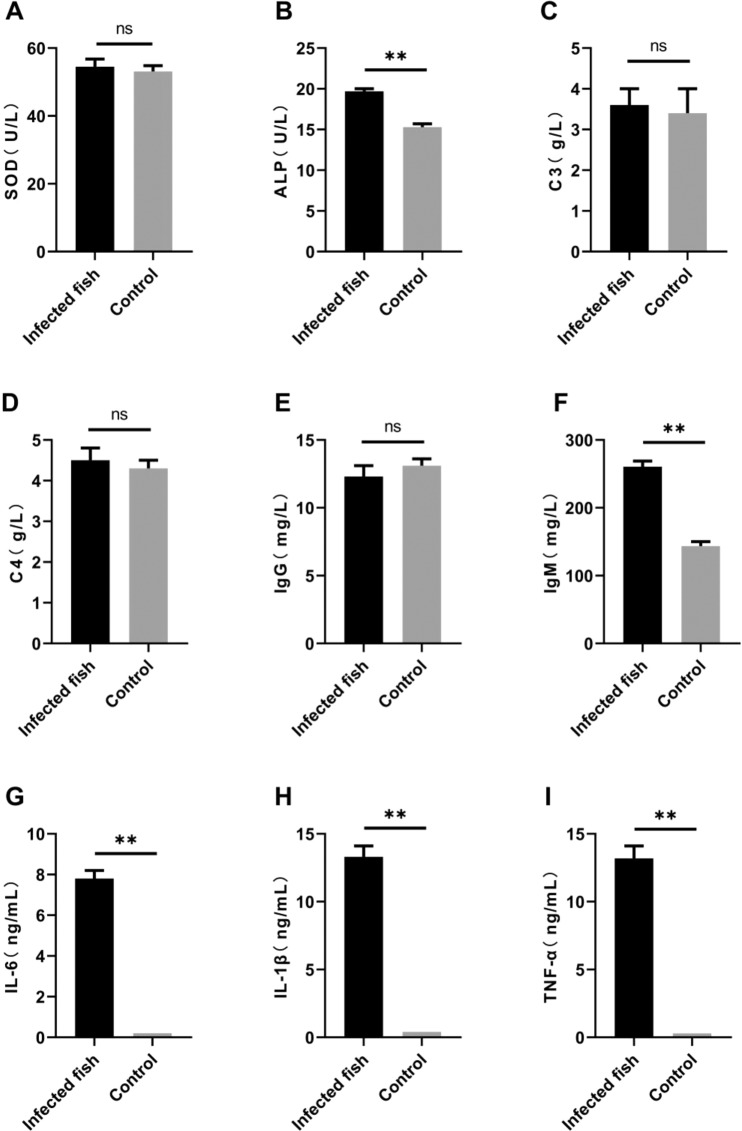
Changes in serum immune indices of infected scattered mirror carp, *Cyprinus carpio*. Non-infected **s**cattered mirror carp from the same pond were used as the control group.

To clarify changes in serum immune markers induced by *T. kitauei*, conventional kits and ELISA were employed to determine antioxidant activity (SOD), alkaline phosphatase, complement C3, complement C4, immunoglobulin IgG and immunoglobulin IgM levels in sera of infected fish, along with cytokines (IL-6, IL-1β and TNF-α) and other indicators (Fig. 7). Notably, differences in the representative indices of antioxidant SOD activity (Fig. 7A), C3 complement (Fig. 7C), C4 complement (Fig. 7D) and immunoglobulin IgG (Fig. 7E) were not significant. Notably, however, immunoglobulin IgM levels were markedly elevated in infected fish (P < 0.01; Fig. 7F). Simultaneously, the alkaline phosphatase content in non-specific indicators was significantly increased in infected fish (P < 0.01) (Fig. 7B), along with levels of cytokines IL-6 (P < 0.01; Fig. 7G), IL-1β (P < 0.01; Fig. 7H) and TNF-α (P < 0.01) (Fig. 7I) in infected fish.

## Discussion

Myxozoans (phylum Myxozoa Grasse) have long been regarded as a class of protozoa, but more recent evidence suggests that they are highly differentiated metazoan parasites that infect invertebrates and aquatic animals as the main hosts^26–28^. Metazoans predominantly infect fish^26^, with limited activity on platyhelminth, reptiles, and amphibians^29,30^. Myxozoans are reported to exert parasitic activity in both organ tissues and cavities^31–35^. *Thelohanellus kitauei* in this study is a typical myxozoan and its parasitic target is the cavity^21^. Since the discovery of the first case of muscle-associated myxozoan parasite in white pelicans from Lake Geneva by Jurine in 1825, more than 2,300 species have been identified^20^. Myxozoans and their toxic effects are currently a worldwide concern. To date, *T. kitauei* infection of fish has only been reported in China^18^, probably due to its specific host being scattered mirror carp (*Cyprinus carpio*). Since carp is the mainstream freshwater aquaculture species in China and there are fewer species of this fish in Europe and the Americas, the main infection host of *T. kitauei* is lacking in these countries. However, the damage inflicted by *T. kitauei* should not be underestimated. In particular, in the central and western regions of China, such infections are extremely common and harmful to fish, leading to considerable economic losses^21,31^. However, due to relatively poor diagnosis and limited basic research on this disease type, few reports are available in the literature. To address this issue, the current study focused on typical *T. kitauei* infection of carp along with the underlying pathological mechanisms and immune response. *T. kitauei* has a strict host and parasitic site range. The hosts include freshwater fish species, such as Israeli and scattered mirror carp, with intestinal tissue identified as the main parasitic site. Our histopathological observations revealed that *T. kitauei* only parasitized the intestinal mucosa of scattered mirror carp and intestinal tissue adjacent to the cysts atrophied due to compression. Only part of the intestinal epithelial tissue remained and inflammation was not obvious. No parasitic tissue was detected, but the peripheral-specific immune response was significantly elevated. Moreover, *T. kitauei* began to proliferate in the basal layer of the intestinal mucosa and formed a large number of cysts with strict fibrous encapsulation. The intestinal epithelial cells ruptured with increasing sac volume, enabling numerous *T. kitauei* to penetrate the intestine and potentially infect the next part or enter the subsequent stage of the life cycle. We speculate that *T. kitauei* causes death of scattered mirror carp through intestinal occlusion by large cysts, which hinders absorption of nutrients, leading to nutritional deficiencies and ultimately, host mortality.

The microscopic and ultramicroscopic characteristics of mature spores and prespore development stages of myxozoans form the main basis for classification of traditional myxozoans and one of the key methods for preliminary identification of clinical isolates. Mature spores of myxozoans are mainly composed of three parts: valve, sporoplasm and polar capsule^1,4,16,20^. *T. kitauei* in this study displayed similar structures in optical and scanning electron microscopy analyses as well as pathological sections. Although the morphological characteristics of mature spores of *T. kitauei* differ from those of other myxozoans, the variations are not significant. However, the pathological changes, infectious mechanisms and immunological responses of *T. kitauei* are very representative. Other reports of myxozoan infections include *Myxobolus cerebralis*^36^, which causes salmon and trout whirling disease, *Tetracapsuloides bryosalmonae*^37^ triggering kidney hyperplasia in carp, *Kudoa thyrsites*^38^ that induces muscle liquefaction in certain marine cultured fish and *Myxobolus turpisrotundus*^39,40^ that leads to loss of retail value of *Carassius auratus gibelio*. Our research showed that *T. kitauei* characteristically infects the submucosa of intestine in carp. This infection appears specific since no reports of other hosts are documented, but further evidence is required.

The immunological performance of *T. kitauei* -infected fish has rarely been studied to date, and even among the reports of diseases caused by myxozoans, few studies have focused on the immunology of myxozoan-related infections. Analysis of the immune response to parasitic infections is a basic approach to understanding the characteristics of parasitic infections and developing effective prevention strategies. Therefore, we assessed a number of immunological indicators of *T. kitauei*-infected carp in the current study. Our experiments disclosed a considerable increase in the total number of white blood cells, lymphocytes and eosinophils in infected fish bodies, but no significant changes in red blood cells. This finding suggests that *T. kitauei* infection stimulates the production of a large number of immune cells. Red blood cells do not participate in the process of immune response during infection and are only responsible for transport of nutrients and oxygen, and therefore, remain relatively unaffected. In natural immunological studies, antioxidant activity and the complement systems of fish did not appear significantly activated. However, alkaline phosphatase activity was markedly elevated during infection, suggesting the involvement of phosphatase in the immune response to parasites. Notably, in terms of specific antibody responses, significant changes were observed for IgM, but not IgG. In response to *T. kitauei* infection, IgM secretion was clearly increased, supporting its involvement in the anti-parasitic immune response. In addition, cytokines participating in specific immune processes, such as IL-6, IL-1β and TNF-α, were significantly elevated, suggesting a critical role of specific immunity following *T. kitauei* infection.

Fish are only intermediate hosts of almost all myxozoans and invertebrates are the terminal host^21^. However, infection of fish, even as intermediate hosts, can cause serious disease and death, resulting in major economic losses. Comprehensive analyses of *T. kitauei* remain to be conducted, including detailed life history, whether infection is related to high-density feeding, the mechanisms underlying disease spread, genomic characteristics, transcriptomics, proteomic features, and more detailed immunological studies. Gradual enrichment of data on these types of pathogens should clarify their pathways of infection and facilitate the development of novel strategies to effectively prevent and control disease outbreaks and their economic impacts.
